# Traumatic Injuries Detected during Post-Mortem Slaughterhouse Inspection as Welfare Indicators in Poultry and Rabbits

**DOI:** 10.3390/ani11092610

**Published:** 2021-09-06

**Authors:** Lenka Valkova, Eva Voslarova, Vladimir Vecerek, Petra Dolezelova, Veronika Zavrelova, Claire Weeks

**Affiliations:** 1 Department of Animal Protection and Welfare and Veterinary Public Health, Faculty of Veterinary Hygiene and Ecology, University of Veterinary Sciences Brno, 612 42 Brno, Czech Republic; H20334@vfu.cz (L.V.); vecerekv@vfu.cz (V.V.); dolezelovap@vfu.cz (P.D.); zavrelovav@vfu.cz (V.Z.); 2 Bristol Veterinary School, University of Bristol, Langford, Bristol BS40 5DU, UK; Claire.Weeks@bristol.ac.uk

**Keywords:** broiler chicken, end-of-lay hen, duck, goose, rabbit, post-mortem inspection, injury

## Abstract

**Simple Summary:**

An analysis of the slaughterhouse post-mortem examination records over a decade showed that for animals transported to slaughter in containers, the risk of traumatic injury was highest in laying hens (2.80%) and rabbits (1.52%), while the overall incidence of trauma was below 0.5% in other species. The results show that the current rearing conditions and/or pre-slaughter handling of poultry and rabbits have comparatively negative welfare consequences, with significantly more traumatic injuries to the limbs than on the trunk in all species studied. In poultry, traumatic findings on the trunk were orders of magnitude lower to negligible, so the focus should be on preventing injuries to the limbs. In rabbits, the difference was less pronounced with a high number of injuries found on both limbs and trunk.

**Abstract:**

The findings of traumatic injuries during post-mortem inspection in slaughterhouses reflect the level of pre-slaughter handling of animals at the farm and during transport to the slaughterhouse. The prevalence of traumatic injuries was monitored in poultry (1,089,406,687 broiler chickens, 20,030,744 laying hens, 1,181,598 turkeys, 37,690 geese, 28,579,765 ducks) and rabbits (1,876,929) originating from farms in the Czech Republic and slaughtered in slaughterhouses in the Czech Republic between 2010 and 2019. The greatest incidence of traumatic injuries was found in laying hens (2.80%) and rabbits (1.52%); while the overall incidence of trauma was less than 0.5% in other species and categories. The results show that the current rearing conditions and/or pre-slaughter handling of poultry and rabbits particularly affect the limbs; traumatic findings were significantly (*p* < 0.01) more frequent on the limbs than on the trunk in all species studied. In poultry, traumatic findings on the trunk were orders of magnitude lower to negligible, so the focus should be on preventing injuries to the limbs. In rabbits, the difference was less pronounced, and many injuries were found on both limbs (0.83%) and trunk (0.69%). Our results emphasize the need to reconsider both housing and pre-slaughter handling methods to determine minimum standards for the protection of rabbits, which are still lacking in European legislation.

## 1. Introduction

Post-mortem injuries on the carcasses of poultry and rabbits detected during inspection at the slaughterhouse reflect the quality of pre-slaughter handling of the animals at the farm and during transport to the slaughterhouse. The advantage of using inspection of slaughtered animals for welfare assessment is the ability to assess injuries individually, on each slaughtered animal, even after removal of feathers or skin, making any lesions that may be commonly overlooked on the farm visible [1]. Traumatic injuries can be evaluated according to where on the body they are located (trunk vs. limbs), their nature (fractures, bruises, abrasions, haematoma) and age (acute or chronic), and thus their origin can be inferred to a certain extent (occurred on farm or during pre-slaughter handling). In addition, each species and category of poultry has its own specific traumatic injuries, mainly because they are reared in different systems and under different conditions [2].

A large proportion of traumatic injuries detected by inspection in slaughterhouses come from pre-slaughter handling. Meluzzi et al. [3] evaluated, in broilers at slaughter, the frequency of haematomas, bruises and fractures that originated from husbandry. The incidence of these traumas was very low, and when they were recorded, they were attributed to handling and transport, not to rearing conditions. Kittelsen et al. [4] also detected more traumas (fractures of limbs, spine, skull) in broilers in slaughterhouses than on farms, which indicates that transport and handling are significant risk factors for trauma. However, pre-slaughter handling is a multi-step process and several factors influence the risk of injury to broilers during this handling [5]. Key risk factors that have been identified include the condition of the bird prior to transport and the method of animal handling [6,7,8,9], the duration of transport and the time of catching [10,11,12], and the stocking density within containers [12]. Most injuries resulting from pre-slaughter handling are found on the wings, and less frequently on the limbs and trunk [8,9,11]. In laying hens, a major risk is posed by pulling end-of-lay hens out of cages [2,13,14] and manually transferring birds into transport containers, where limb injuries are most likely to occur [14,15]. Osteoporosis is also an important predisposing factor, leading to fractures of the limbs and keel bone due to demineralization and increased bone fragility and brittleness [16]. In waterfowl, the limbs are weak relative to gallinaceous poultry, and so careless handling poses an increased risk of trauma [17]. Because of their high weight, turkeys must not be manually carried by their limbs with their heads down but must be supported under the body. Furthermore, handling of poultry immediately prior to slaughter, such as hanging and stunning, contributes to the frequency of traumatic injuries, especially to the limbs [18,19]. Gaseous stunning methods that render birds unconscious before hanging minimize injuries sustained from wing flapping [20,21,22]. 

Rabbits are also transported to slaughterhouses in containers. The high number of traumatic findings in rabbits reported by Valkova et al. [23] corresponds to the high level of mortality during transport to slaughterhouses [24]. Even for rabbits, the handling of animals during catching, loading and unloading from transport containers can be considered as risk factors in terms of injury or even death. This risk increases if handling is careless, and if large numbers of animals are transported in a single shipment [25,26]. The cause lies in the human factor, as staff caution decreases when handling large numbers of animals [26]. The attitude of the staff towards the animals plays a key role in the handling of slaughter rabbits [27]. Rough handling leads to traumatic lesions such as bruises, abrasions, and/or fractures [28]. Fighting due to mixing of animals during loading can also cause injuries, but this is more likely to be the case for older animals culled from breeding and is not a problem for fattening rabbits transported to slaughter while still pre-pubertal [29]. 

In order to list the aetiological factors that contribute to the findings of traumatic injuries in slaughterhouses, it is necessary to mention the factors that originate in the husbandry of the animals in question. Both very high stocking density and low stocking density may lead to weaker birds being victims of injurious attacks causing trauma such as bruising [30,31]. Furthermore, it is advisable to provide waterfowl with access to water, an appropriate light regime and good quality litter. Good litter quality is of utmost importance for all poultry species, otherwise there is an increased incidence of painful foot pad dermatitis. Increased lying as a consequence may lead to bruises on the pectoral muscles. The housing system has a major impact on the incidence of injuries in laying hens. A number of studies have described differences in the fitness and health of laying hens between birds housed in cages or alternative housing systems e.g., [32,33,34,35,36,37] and Weeks et al. [38] also linked this to the fitness for transport of laying hens. The housing system also affects the incidence of injuries in rabbits. Rabbits kept in cage systems have more traumatic injuries than those kept in pens [39]. Other factors affecting the incidence of injuries on farms include high stocking densities [40] and insufficiently enriched environments [41,42]. The wire netting floor commonly used for housing rabbits can cause foot pad injuries, but in fattening rabbits, the problem does not usually develop owing to the shorter production cycle [27].

The aim of this study was to assess the welfare of poultry (end-of-lay hens, broiler chickens, turkeys, geese, ducks) and rabbits in relation to the prevalence of traumatic findings detected post-mortem at the slaughterhouse. The incidence of traumatic injuries was further evaluated in the categories and species of animals studied with respect to their localization (limbs and trunk).

## 2. Materials and Methods

The welfare of poultry and rabbits slaughtered in slaughterhouses was assessed in terms of the prevalence of traumatic lesions in animals detected during veterinary post-mortem inspection in slaughterhouses in the Czech Republic during the ten-year period from 2010 to 2019.

The Czech Republic is a landlocked country in Central Europe with an estimated population of 10,6 million, about three-fourths of the population being urban. The Czech Republic has a hilly landscape that covers an area of 78,871 square kilometers with a temperate climate. The contribution of agriculture to the GDP in the Czech Republic is about average for the EU. Plant production predominates over animal production in the Czech Republic. Milk production forms almost one half of the total animal output. The highest portion of animal farming is pig farming, followed by cattle, which are approximately on the same level as poultry. Egg production accounts for 5% of the total animal production. About 80% of egg-laying hens in commercial production in the Czech Republic are kept in enriched cages. Meat rabbits are almost exclusively housed in cages. As a member state of the EU, the Czech Republic is obliged to follow EU animal welfare legislation. However, some stricter measures have been maintained or established, namely a ban on force-feeding of ducks and geese, a maximum 8-hour journey limit for animals transported for the purpose of being slaughtered and a requirement of secondary education in the field of butchery for persons carrying out slaughter of animals in the slaughterhouse.

The prevalence of traumatic injuries was monitored retrospectively in poultry and rabbits originating from farms in the Czech Republic and slaughtered in slaughterhouses in the Czech Republic. In the period under review, a total of 1,089,406,687 broilers, 20,030,744 laying hens, 1,181,598 turkeys, 37,690 geese, 28,579,765 ducks and 1,876,929 rabbits were slaughtered in slaughterhouses in the Czech Republic. Only animals that were slaughtered for human consumption and thus underwent slaughterhouse veterinary inspection were included in the analysis. Animals that died on farms or during transport were not included in the analysis.

The assessment of traumatic injuries in animals was carried out by official veterinarians of the State Veterinary Administration, who recorded the number of traumatic findings. Inspections were carried out after slaughter and defeathering or depelting. The classification of findings was based on the methodology for postmortem examinations of animals at slaughterhouses. All animals slaughtered at slaughterhouses (their carcasses) are inspected by certified veterinary inspectors who received uniform training and certification of competence to perform this kind of veterinary slaughterhouse inspections. The legal requirements and specification of veterinary postmortem examination are laid down by European Union legislation [43]. The results of slaughterhouse veterinary examinations were collected in the central database of the State Veterinary Administration, from which data were then extracted for analysis.

During postmortem examinations, veterinary inspectors differentiated between injury to the body (including keel bone fractures) and injury to the limbs (including wings). Wounds at various stages of healing in skin and underlying tissues, haematoma in the hypodermis and muscles, bruises, dislocations, fractures (open and closed) and other changes that may arise as a consequence of the housing system used, incorrect handling and interactions between animals on the farm, during transport or during lairage before slaughter were included among findings of trauma. However, veterinary inspectors did not aim to classify the cause of the antemortem injuries. They only distinguished injuries that occurred ante- and postmortem on the basis of their appearance (observation of biological processes related to tissue regeneration, presence of clotting, swelling, inflammation, scarring). Postmortem injuries (technology-related damage following stunning) were not included among findings of trauma analyzed in our study.

For the purposes of the study, the prevalence of traumatic injuries to the trunk and limbs in poultry (end-of-lay hens, broiler chickens, turkeys, geese, ducks) and in rabbits relative to the total number of birds and rabbits slaughtered was analyzed. Differences in the prevalence of traumatic injuries between limbs and trunk within each species, and differences in the prevalence of traumatic injuries in limbs and trunk between species were compared. In domestic chickens (*Gallus gallus*), differences between adult (end-of-lay hens) and meat birds (broiler chickens) were also assessed. For other species, the difference between adult and fattened animals was not evaluated, because adult animals other than laying hens are not commonly slaughtered for human consumption in slaughterhouses in the Czech Republic.

The results were statistically evaluated using the statistical package Unistat v. 6.5. (Unistat Ltd., London, UK). Differences in frequency of traumatic injuries among various species/categories of animals and differences in frequency of traumatic injuries to the limbs and trunk in the monitored animal species and categories were tested on the basis of a chi-square test within the r × c and 2 × 2 contingency table procedures [44]. In the case of 2 × 2 contingency table, Yates´ correction was used throughout the computation. When the frequencies in the contingency table were lower than 5, a Fisher exact test was used instead of a Chi-square test [44]. 

## 3. Results

The results show that the highest number of traumatic injuries was found in laying hens (2.7957%) and rabbits (1.5184%), while the lowest number of traumatic injuries was found in ducks (0.0031%). In domestic chickens, there was also a difference (*p* < 0.01) between adult (end-of-lay hens) and fattened birds (broiler chickens) in both the number of traumatic findings on the limbs and the number of traumatic findings on the trunk. In both cases, a significantly higher (*p* < 0.01) number of injuries were recorded for laying hens than broiler chickens. 

The incidence of traumatic injuries to the limbs and trunk in different species and categories of poultry and rabbits is provided in Table 1. There was a statistically significant (*p* < 0.01, Χ^2^ = 23,894,554.69, df = 5) difference in the number of traumatic findings on the limbs between all species (Table 2). The highest number of traumatic injuries to the limbs was found in laying hens (2.7844%) and the lowest in ducks (0.0029%). In the interspecies comparison of the number of traumatic findings on the body, there was a statistically significant (*p* < 0.01, Χ^2^ = 1,010,924.32, df = 5) difference between all species except laying hens and geese, and geese and broiler chickens (Table 3). The highest number of traumatic findings on the body was found in rabbits (0.6849%) and the lowest in ducks (0.0001%).

**Figure 1:**
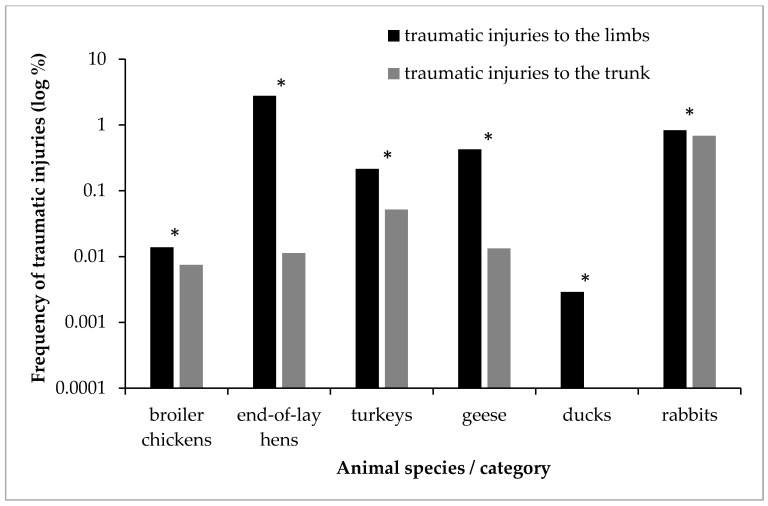
Comparison of frequency (log %) of traumatic injuries to limbs and trunk in different species and categories of poultry and rabbits. * statistically significant difference between frequency of traumatic injuries to the limbs and to the trunk in the given species/category (*p* < 0.01).

A comparison of the frequency of traumatic injuries to the limbs and body in different species and categories of poultry and rabbits is shown in Figure 1. For better visual representation, the values on the *y*-axis were converted to a logarithmic scale. In all species studied, traumatic findings on the limbs were more frequently statistically significantly (*p* < 0.01) than on the trunk (Table 4).

## 4. Discussion

The overall incidence of trauma varied considerably between the poultry and rabbits slaughtered. Very low values were seen in ducks and levels were comparatively low in broilers (0.01%), whereas the heavier meat birds (geese and turkeys) sustained more damage. Substantially more trauma was recorded for end-of-lay hens and rabbits. Levels of injury are seldom reported in the literature but Gerpe et al. [14] recorded a rate of severe injury of 8.1% during catching and crating of end-of-lay hens from aviaries, which is substantially more than our findings for hens removed predominantly from the enriched cage system. A predisposing factor in cage housing may be the removal of hens from their cages, where numerous injuries may occur when hens are dragged through the cage opening, with furniture such as perches an additional hazard [2]. Although Knowles and Wilkins [15] recommended catching by 2 legs and the use of breast slides over the feed trough to reduce injuries, the speed of commercial depopulation means this is seldom practiced. In the Czech Republic, most laying hens are still kept in cages; a ban on cage housing will become effective from 2027.

The records analyzed in our study could not distinguish between injuries originating on the farm and those occurring during transport or at the slaughterhouse. However, evidence suggests that pre-slaughter handling of animals is the main factor influencing the occurrence and frequency of traumatic injuries [3,4]. Assuming that the dominant origin of injuries is during transport operations, this finding is consistent with the results of studies also highlighting high mortality during transport to slaughterhouses, both in laying hens [45,46] and rabbits [24]. In end-of-lay hens, this may be attributed to their lower market value [47] and the corresponding lower care and rougher handling [46] not respecting the fragility of the bones of end-of-lay hens [16]. However, Weeks et al. [38] note the scope for improvement whereby a slaughter plant operating risk assessments for each consignment of end-of-lay hens, and adjusting procedures accordingly, routinely achieved median DOAs of 0.13% even for journeys exceeding 800 km. Rabbits are the species for which animal welfare and species-specific needs are the most overlooked in the European Union owing to the absence of specific legislation for mandatory minimum requirements for the protection of rabbits in agriculture in the majority of Member States [25,48]. For both species, the small number of establishments available for slaughter of rabbits and end-of-lay hens leading to longer transport times may also play a role.

Our comparison of the position of traumatic injuries in poultry and rabbits post mortem shows that traumatic injuries to the limbs occurred significantly more often than traumatic injuries to the trunk in all species studied. This can be related to pre-slaughter handling, which damages the limbs more than the trunk of the animals, as the limbs are also the main part of the body by which poultry and rabbits are caught and carried when handled during catching, loading, unloading and hanging at the slaughterhouse [2,5,26]. This places significantly more stress on the limbs and thus exposes them to the risk of traumatic injury (abrasions, wounds, bruises, sprains, dislocations and/or fractures) than the more volumetrically compact animal trunks. Other studies have shown that especially the wings of birds are often damaged. Jacobs et al. [11] found that catching at the farm had a significant effect on the incidence of wing fractures in broilers (the difference in prevalence at the farm and after catching was 0.12% and 1.88%, respectively, and rose to a mean of 1.95% post mortem, which could include machine damage). In addition, when evaluating the factors that influenced the incidence of bruising in broilers at slaughterhouses, Saraiva et al. [12] and Langkabel et al. [8] observed the most bruising on wings. On the other hand, Gouveia et al. [49] observed more bruising on the body (pectoral musculature) than the legs and/or wings, which was probably due to increased injuries caused by untrained personnel on the openings of transport containers. At this stage of transport, we may include the method of catching among the risk factors. Automated loading of birds into transport containers appears to be less traumatic than manual methods [6,7]. In manual catching, lifting by the wings and holding under the body in an upright position is preferable to carrying by the limbs with the head down [9]. Other risk factors include duration of catching and time of the day of the catching, where positive correlation was found between the prevalence of wing fractures and duration of catching and loading, and the risk of bruising also increased when birds were loaded at night [11]. Conversely, Saraiva et al. [12] observed a higher percentage of bruising when birds were caught before midnight and Nijdam et al. [10] observed more bruising in broilers transported during the day than at night. The process of hanging and stunning increases the prevalence of traumatic wing injuries due to wing flapping and consequent wing injuries [18,19].

When comparing traumatic injuries between broiler chickens and laying hens, we found a statistically significant difference both in the limbs (0.014% and 2.784%, respectively) and in the trunk (0.008% and 0.011%, respectively). Not only the period of intensive egg production but also the housing system has an impact on the quality and strength of the musculoskeletal system of poultry. Cage systems in which laying hens are still predominantly housed in the Czech Republic limit the movement of laying hens, and thus increase their susceptibility to the development of disuse osteoporosis [35]. Aviary housing systems, where there is greater opportunity for active movement, have been shown to improve musculoskeletal health [34]. The level of health, fitness and recovery capacity also have an impact, with that being higher in younger animals (broilers) than in the older ones (laying hens) that are depleted by intensive egg production [45]. If we assume that traumatic injuries are predominantly associated with catching the animals and their transport to slaughter, then the quality of the bones and condition of the birds is crucial and may account for much of the differences in numbers of injuries observed in end-of-lay hens and broilers. However, the very low and sometimes negative value of end-of-lay hens is undoubtedly a factor, whereas broilers are high-value birds for human meat consumption where damage to the carcass carries financial penalties.

In laying hens, traumatic limb injuries were the most frequent (2.78%) compared to other species and categories studied. Rabbits ranked second in terms of frequency of limb trauma (0.83%) but also in terms of total number of traumas irrespective of location (1.52%). In addition, rabbits were found to have the highest incidence of trunk traumatic injuries, namely 0.69%, while the incidence of trunk trauma was an order of magnitude lower in poultry, ranging from 0.00% (ducks) to 0.05% (turkeys). When being unloaded, rabbits are manually removed from cages, carried and placed in transport containers. Verga et al. [28] found that when rabbits were loaded into transport containers placed on the transport vehicle, the prevalence of loin bruising was reduced by 0.44% compared to their loading into the transport containers inside the farm and then transferring to the transport vehicle in the transport containers. Bruising was most frequent on the limbs, chest and loins, which is consistent with our findings where injuries were frequently found on both the limbs and the trunk in rabbits. In terms of injuries, not only the pre-slaughter handling but also the rearing method is risky for rabbits. In the Czech Republic, rabbits are almost exclusively kept in cages, where limbs are subject to bruising and abrasions caused by cage mesh. Rabbits live in battery cages on bare wire mesh floors, which is a frequent cause of bruises and injuries (paracetosis, foot pad dermatitis), so it is advisable to cover them at least with mats [50]. Of all the farming systems used, cage rearing is the least suited to the behavioral needs of this species. The vast majority of rabbits are born in a cage and remain in one until they are taken to the slaughterhouse [51]. Inappropriate cage dimensions limit the possibilities of movement and other natural activities and postures, which, among other things, leads to abnormal skeletal development in rabbits (bone deformities, hypoplasia of bone tissue) and facilitates the incidence of fractures [52]. European legislation has not yet set any minimum standards for the design, size and equipment for rabbit breeding, fattening and/or transport facilities. Moreover, while there is increasing pressure to abandon cage rearing for poultry, and in several countries this ban has already come into force or will come into force in the coming years, this is not yet the case for rabbits. The results of our study show that current rearing and/or transport conditions lead to a high incidence of injury in rabbits relative not only to poultry, but also to other livestock species [53].

Geese had the third highest incidence of traumatic injuries, predominantly to their limbs (0.43%), while injuries to the trunk were rarely detected (0.01%). Similarly, for turkeys, limb injuries (0.21%) significantly outnumbered trunk injuries (0.05%). A possible explanation for this is the larger size of geese and turkeys relative to transport cages compared to the much smaller broiler chickens and ducks, which had the lowest incidence of injuries (0.02% and 0.00%, respectively). Larger animals may experience more frequent traumatic injury to limbs performing escape behaviors during loading into and unloading from transport containers. When comparing different handling methods for loading turkeys into transport containers, the method of herding turkeys to the loading site and having them self-board using a ramp into a transport container placed on the transport vehicle was assessed to be the least stressful and cause the least injuries, compared to manually carrying and pushing them into transport containers [54].

The limitations of the study include inability to quantify risk factors leading to injuries as the extent of data collected during routine post-mortem slaughterhouse inspections and archived on the national level is limited, i.e., more thorough analysis of the origin of traumatic injuries recorded and the origin of injured birds was not possible. However, since all birds and rabbits slaughtered in the Czech slaughterhouses over a ten-year period were included in the study, the results show overall trends concerning prevalence of traumatic injuries in slaughtered poultry and rabbits reared in intensive housing systems.

## 5. Conclusions

An analysis of the slaughterhouse post-mortem examination data recorded over a decade showed that the risk of traumatic injury was highest in laying hens (2.80%) and rabbits (1.52%), while the overall incidence of trauma was below 0.5% in other species. The results show that the current rearing conditions and/or pre-slaughter handling of poultry and rabbits have negative welfare consequences, with significantly more traumatic injuries to the limbs than on the trunk in all species studied. In poultry, traumatic findings on the trunk were orders of magnitude lower to negligible, so the focus should be on preventing injuries to the limbs. In rabbits, the difference was less pronounced with a high number of injuries found on both limbs and trunk. Our results thus confirm the need to re-assess the farming methods and pre-slaughter handling used and ideally to legislate minimum standards ensuring the protection of rabbits, which are still lacking in European legislation (despite rabbits being in the sixth position regarding the numbers of farmed animals killed for human consumption in the EU). More detailed post-mortem inspection carried out routinely in the slaughterhouses would be beneficial not only to identify specific risk factors in general but also to compare individual farmers and transporters, i.e., to exercise closer scrutiny when the number of injured animals exceeds some level.

## Figures and Tables

**Table 1:** The incidence of traumatic injuries to the limbs and trunk in poultry and rabbits as detected during slaughterhouse inspection in the Czech Republic from 2010 to 2019.

Species/ Category	Number of Animals Slaughtered	Number of Traumatic Injuries to the Limbs	Percentage of Traumatic Injuries to the Limbs (%)	Number of Traumatic Injuries to the Trunk	Percentage of Traumatic Injuries to the Trunk
End-of-lay hens	20,030,744	557,726	2.7844 ^a^	2269	0.0113 ^x^
Broiler chickens	1,089,406,687	150,545	0.0138 ^e^	81,770	0.0075 ^y^
Turkeys	1,181,598	2532	0.2143 ^d^	612	0.0518 ^w^
Geese	37,690	160	0.4232 ^c^	5	0.0133 ^x,y^
Ducks	28,579,765	832	0.0029 ^f^	42	0.0001 ^z^
Rabbits	1,876,929	15,645	0.8335 ^b^	12,855	0.6849 ^v^

^a–f^ percentages in the column with different superscripts differ (*p* < 0.01), ^v–z^ percentages in the column with different superscripts differ (*p* < 0.01).

**Table 2:** The pairwise comparisons (*p*-value) of frequency of traumatic injuries to the limbs in monitored animal species and categories.

Species/Category					
	End-of-lay hens				
Broiler chickens	0.0000	Broiler chickens			
Turkeys	0.0000	0.0000	Turkeys		
Geese	0.0000	0.0000	0.0000	Geese	
Ducks	0.0000	0.0000	0.0000	0.0000	Ducks
Rabbits	0.0000	0.0000	0.0000	0.0000	0.0000

**Table 3:** The pairwise comparisons (*p*-value) of frequency of traumatic injuries to the trunk in monitored animal species and categories.

Species/Category					
	End-of-lay hens				
Broiler chickens	0.0000	Broiler chickens			
Turkeys	0.0000	0.0000	Turkeys		
Geese	0.7239	0.1968	0.0011	Geese	
Ducks	0.0000	0.0000	0.0000	0.0000	Ducks
Rabbits	0.0000	0.0000	0.0000	0.0000	0.0000

**Table 4:** The pairwise comparisons (*p*-value) of frequency of traumatic injuries to limbs and trunk in monitored animal species and categories.

Species/Category					
End-of-lay hens	Broiler chickens	Turkeys	Geese	Ducks	Rabbits
0.0000	0.0000	0.0000	0.0000	0.0000	0.0000

## Data Availability

Data for analysis was obtained from the information system of the State Veterinary Administration of the Czech Republic. The datasets generated and analysed during the current study are available from the corresponding author on reasonable request.
